# Correction: Impact of COVID-19 vaccination on mortality after acute myocardial infarction

**DOI:** 10.1371/journal.pone.0314647

**Published:** 2024-11-22

**Authors:** Mohit D. Gupta, Shekhar Kunal, Girish M. P., Dixit Goyal, Rajeev Kumar Malhotra, Prashant Mishra, Mansavi Shukla, Aarti Gupta, Vanshika Kohli, Nitya Bundela, Vishal Batra, Ankit Bansal, Rakesh Yadav, Jamal Yusuf, Saibal Mukhopadhyay

There are errors in the Vaccinated and Unvaccinated values in Table 1. The Vaccinated value for 18–39 yrs under the Sex variable should have been 112(10.3%). In the Co-morbidities variable, the correct Unvaccinated value of Family History should be 120(24.4%) and in the Revascularization the Vaccinated and Unvaccinated values should be 139(12.1%) and 53 (10.6%) respectively. Please see the correct Table 1 here.

**Table 1:** Baseline Patients Characteristics of vaccinated and unvaccinated AMI patients

Variable	Vaccinated (n = 1086)	Unvaccinated (n = 492)	P-value (Unweighted)	P-value (Weighted)
**Sex**				
Male	895(82.4%)	378(76.8%)	0.009	0.944
Female	191(17.6%)	114(23.2%)		
Age, Median (IQR), yrs	55[46–62]	56[48–65]	<0.001	0.939
18–39 yrs	112(10.3%)	33(6.7%)		
40–64 yrs	760(70.0%)	325(66.1%)		
>65 yrs	214(19.7%)	134(27.2%)		
**Co-morbidities**				
Diabetes	233(21.5%)	114(23.2%)	0.446	0.924
Hypertension	347(32.0%)	156(31.7%)	0.923	0.992
COPD	35 (3.2%)	15 (3.0%)	0.854	
CKD	03 (0.27%)	01 (0.20%)	0.789	
Heart failure	30 (2.7%)	18 (3.6%)	0.336	
Dyslipidaemia	42(3.9%)	24(4.9%)	0.353	0.961
Smoking	474(43.6%)	184(37.4%)	0.020	0.958
Physical Activity	484(44.6%)	217(44.1%)	0.864	
Family History	261(24.0%)	120(24.4%)	0.878	
Revascularization	139 (12.1%)	52 (10.6%)	0.208	0.894

**Abbreviations:** AMI: acute myocardial infarction; COPD: chronic obstructive pulmonary disease; CKD: chronic kidney disease; IQR: inter-quartile range; yrs: years

S1 Table is uploaded incorrectly. Please view the correct S1 Table below.

## Supporting information

S1 Table Comparison of patients’ characteristics between 30 days outcome. (DOCX)
