# Supplementary material for: Impact of COVID-19 vaccination on mortality after acute myocardial infarction
Source: PLoS One. 2023 Sep 1;18(9):e0291090. doi: 10.1371/journal.pone.0291090 (PMC10473468; doi:10.1371/journal.pone.0291090)
Supplement: S2 Table — (DOCX) [file pone.0291090.s002.docx]

**Supplementary table 2: Propensity score weighted logistic regression models for assessing the odds ratio of vaccination on mortality during one - six months of follow-up**

|  | **Mortality between 1 - 6 months (n=75)** | | | |
| --- | --- | --- | --- | --- |
| **Variable** | **Vaccinated (n=32)** | **Unvaccinated (n=43)** | **Adjusted Odds Ratio (95% CI)** | **P-value** |
| Overall | 32 | 43 | 0.34 [0.24-0.48] | **<0.001** |
| Sub-group Analysis | | | | |
| Male | 28 | 27 | 0.48 [0.32-0.70] | **<0.001** |
| Female | 4 | 16 | 0.12 [0.05-0.26] | **0.0003** |
| Age | | | | |
| 18-39 | 2 | 1 | 0.55 [0.22-1.08] | 0.151 |
| 40-64 | 21 | 25 | 0.37 [0.23-0.52] | **<0.001** |
| >=65 | 9 | 17 | 0.29 [0.15-0.55] | **<0.001** |
| Diabetes | | | | |
| Yes | 4 | 8 | 0.24 [0.09-0.57] | **0.003** |
| No | 28 | 35 | 0.36 [0.24-0.52] | **<0.001** |
| Hypertension | | | | |
| Yes | 10 | 17 | 0.28 [0.15-0.51] | **0.004** |
| No | 22 | 26 | 0.38 [0.25-0.58] | **<0.001** |
| Hyperlipidaemia | | | | |
| Yes | 0 | 3 | Cannot be computed due to zero counts | |
| No | 32 | 40 | 0.37 [0.26-0.52] | **<0.001** |
| Smoking | | | | |
| Smoker | 11 | 14 | 0.28 [0.15-0.51] | **<0.001** |
| Non-smoker | 21 | 29 | 0.38 [0.24-0.58] | **<0.001** |
| Physical Activity | | | | |
| Yes | 22 | 18 | 0.50 [0.31-0.79] | 0.3789 |
| No | 10 | 25 | 0.20 [0.11-0.35] | **<0.001** |
| Family History | | | | |
| Yes | 10 | 6 | 1.09 [0.51-2.33] | 0.832 |
| No | 22 | 37 | 0.26 [0.17-0.38] | **<0.001** |
