# Supplementary material for: Impact of COVID-19 vaccination on mortality after acute myocardial infarction
Source: PLoS One. 2023 Sep 1;18(9):e0291090. doi: 10.1371/journal.pone.0291090 (PMC10473468; doi:10.1371/journal.pone.0291090)
Supplement: Attachment — Submitted filename: Response to Reviewers.docx [file pone.0291090.s003.docx]

**Comments to the Author**

1. Is the manuscript technically sound, and do the data support the conclusions?

The manuscript must describe a technically sound piece of scientific research with data that supports the conclusions. Experiments must have been conducted rigorously, with appropriate controls, replication, and sample sizes. The conclusions must be drawn appropriately based on the data presented.

Reviewer #1: Yes

Reply: We thank the learned reviewer for the comments.

2. Has the statistical analysis been performed appropriately and rigorously?

Reviewer #1: Yes

Reply: We thank the learned reviewer for the comments.

3. Have the authors made all data underlying the findings in their manuscript fully available?

The [PLOS Data policy](http://www.plosone.org/static/policies.action#sharing) requires authors to make all data underlying the findings described in their manuscript fully available without restriction, with rare exception (please refer to the Data Availability Statement in the manuscript PDF file). The data should be provided as part of the manuscript or its supporting information, or deposited to a public repository. For example, in addition to summary statistics, the data points behind means, medians and variance measures should be available. If there are restrictions on publicly sharing data—e.g. participant privacy or use of data from a third party—those must be specified.

Reviewer #1: Yes

4. Is the manuscript presented in an intelligible fashion and written in standard English?

PLOS ONE does not copyedit accepted manuscripts, so the language in submitted articles must be clear, correct, and unambiguous. Any typographical or grammatical errors should be corrected at revision, so please note any specific errors here.

Reviewer #1: Yes

Reply: We thank the learned reviewer for the comments.

5. Review Comments to the Author

Please use the space provided to explain your answers to the questions above. You may also include additional comments for the author, including concerns about dual publication, research ethics, or publication ethics. (Please upload your review as an attachment if it exceeds 20,000 characters)

Reviewer #1: At the outset, I would like to congratulate the authors for having taken up this study. In this study, the authors have evaluated two COVID-19 vaccines (COVISHIELD, COVAXIN) available in India. The primary outcome of the study was all-cause mortality both at one month and at six months of follow-up. The authors conclude that COVID-19 vaccines showed a decrease in all-cause mortality at 30 days and six months following AMI.

It is a very elegant study but it must be kept in mind that this is a single centre retrospective observational study.

A few questions for the authors:
• It is not mentioned how many participants received single dose and how many received two doses of vaccine.

Reply: We thank the learned reviewer for the comments. We have now highlighted in our MS regarding the number of participants receiving single and two doses of COVID-19 vaccine.

• Why the effect of third dose (booster)of COVISHIELD not studied in this study?

Reply: We thank the learned reviewer for the comment. The data included in this study was before the launch of the booster dose of COVID-19 vaccines in India and hence enrolled patients had not received the booster dose.

• Why only one parameter that is all-cause mortality was evaluated in this study?

Reply: We thank the learned reviewer for the comment. We had included all-cause mortality as one of the hard end-points for our study. In a resource limited developing country such as India during the COVID-19 pandemic, it was not possible to collect data regarding all other end points. This has been highlighted as one of the limitations of our study.

6. PLOS authors have the option to publish the peer review history of their article ([what does this mean?](https://journals.plos.org/plosone/s/editorial-and-peer-review-process#loc-peer-review-history)). If published, this will include your full peer review and any attached files.


If you choose “no”, your identity will remain anonymous but your review may still be made public.


**Do you want your identity to be public for this peer review?** For information about this choice, including consent withdrawal, please see our [Privacy Policy](https://www.plos.org/privacy-policy).

Reviewer #1: No
