# Supplementary material for: Impact of COVID-19 vaccination on mortality after acute myocardial infarction
Source: PLoS One. 2023 Sep 1;18(9):e0291090. doi: 10.1371/journal.pone.0291090 (PMC10473468; doi:10.1371/journal.pone.0291090)
Supplement: Attachment — Submitted filename: Reply to reviewers comments.docx [file pone.0291090.s004.docx]

**Reviewers' comments:**

Reviewer's Responses to Questions

Comments to the Author

1. If the authors have adequately addressed your comments raised in a previous round of review and you feel that this manuscript is now acceptable for publication, you may indicate that here to bypass the “Comments to the Author” section, enter your conflict of interest statement in the “Confidential to Editor” section, and submit your "Accept" recommendation.

Reviewer #2: All comments have been addressed

Reviewer #3: All comments have been addressed

Reviewer #4: (No Response)

Authors reply: We thank the learned reviewers for the comments.

2. Is the manuscript technically sound, and do the data support the conclusions?

The manuscript must describe a technically sound piece of scientific research with data that supports the conclusions. Experiments must have been conducted rigorously, with appropriate controls, replication, and sample sizes. The conclusions must be drawn appropriately based on the data presented.

Reviewer #2: Yes

Reviewer #3: No

Reviewer #4: (No Response)

Authors reply: We thank the learned reviewers for the comments.

3. Has the statistical analysis been performed appropriately and rigorously?

Reviewer #2: Yes

Reviewer #3: No

Reviewer #4: (No Response)

Authors reply: We thank the learned reviewers for the comments.

4. Have the authors made all data underlying the findings in their manuscript fully available?

The PLOS Data policy requires authors to make all data underlying the findings described in their manuscript fully available without restriction, with rare exception (please refer to the Data Availability Statement in the manuscript PDF file). The data should be provided as part of the manuscript or its supporting information, or deposited to a public repository. For example, in addition to summary statistics, the data points behind means, medians and variance measures should be available. If there are restrictions on publicly sharing data—e.g. participant privacy or use of data from a third party—those must be specified.

Reviewer #2: Yes

Reviewer #3: No

Reviewer #4: (No Response)

Authors reply: We thank the learned reviewers for the comments.

5. Is the manuscript presented in an intelligible fashion and written in standard English?

PLOS ONE does not copyedit accepted manuscripts, so the language in submitted articles must be clear, correct, and unambiguous. Any typographical or grammatical errors should be corrected at revision, so please note any specific errors here.

Reviewer #2: Yes

Reviewer #3: No

Reviewer #4: (No Response)

Authors reply: We thank the learned reviewers for the comments.

6. Review Comments to the Author

Please use the space provided to explain your answers to the questions above. You may also include additional comments for the author, including concerns about dual publication, research ethics, or publication ethics. (Please upload your review as an attachment if it exceeds 20,000 characters)

Reviewer #2: Very elegant manuscript. Well written and well structured, with a valid hypothesis. Conclusions should be interpreted with caution, with this only being a single centre study.

Authors reply: We thank respected reviewer for kind words of appreciation. We agree that all the results have to be interpreted with caution. We have highlighted very clearly in the manuscript that this is a single centre study and results have to be interpreted with caution.

Reviewer #3: - missing base line characteristics as COPD, heart failure, CKD, are needed.

- no laboratory or imaging data were included.

Authors reply: We thank the learned reviewers for the comments. We have now added the data for missing baseline characteristics in the MS (Table 1). Since this was a retrospective review to determine any causality between COVID-19 vaccination and AMI, laboratory and imaging data were not included.

- missing other variables as type of AMI (Stemi or NSTEMI) would affect the MACE

Authors reply: We thank the learned reviewers for the comments. We had categorically looked into occurrence of STEMI following COVID-19 vaccination which we had mentioned in the methods. We sought to study the most severe form of MI after vaccination. Since this was a retrospective review carried out during the pandemic in a resource limited set-up, all comer STEMI cases were included.

- the method and timing of revascularization would significantly affect the study results.

Authors reply: We thank the learned reviewers for the comments. The details of revascularization with PCI have now been included in the manuscript. The figure (Figure 3) and table (table 1) have been modified. There was no impact of revascularization on the protective effect of COVID-19 vaccination. This has now been added in the MS.

Reviewer #4: We have some comments:

1- The authors should defend the rationale on why including only STEMI ACS,

Authors reply: We thank the learned reviewers for the comments. This study was a retrospective analysis of effect of vaccination on STEMI patients. This is the most severe form of coronary artery disease and hence we aimed to see the effect of vaccination on mortality after STEMI.

2- In the methodology and to have clear inclusion criteria, the authors should determine the maximum and minimum time interval between vaccination day and occurence of STEMI event needed for enrollment.

Authors reply: Learned Reviewer have very rightly suggested the mention of time interval. However, we must state that the effect of vaccination on STEMI and the time period after which it may or may not effect is unknown. Hence, we didn’t define any time interval in inclusion, rather we included all the patients who were vaccinated and studied the effect of vaccination in time quartiles to clearly see the time interval when these events occur.

3- All cause mortality is a complex outcome with grand multifactorial confounders and is difficult to analyse. The authors should defend and discuss this point properly.

Authors reply: Learned Reviewer has very rightly pointed out the limitation of using all cause mortality. However, we must state that all cause mortality is a hard end point and we have tried to adjust all the possible confounders in our analysis. In this study during covid times, it was extremely challenging to collect data of all possible outcomes in a resource limited country like India. The same has now been included in the discussion
