# Supplementary material for: Correction: Impact of COVID-19 vaccination on mortality after acute myocardial infarction
Source: PLoS One. 2024 Nov 22;19(11):e0314647. doi: 10.1371/journal.pone.0314647 (PMC11584104; doi:10.1371/journal.pone.0314647)
Supplement: S1 Table — (DOCX) [file pone.0314647.s001.docx]

**Supplementary table 1: Comparison of patients’ characteristics between 30 days outcome**

| **Variable** | **Mortality (n=201)** | **Alive (n=1377)** | **P-value** |
| --- | --- | --- | --- |
| **Sex** |  |  |  |
| Male | 145(72.1%) | 1128(81.9%) | **0.001** |
| Female | 56(27.9%) | 249(18.1%) |  |
| Age, Median(IQR), yrs | 55[46-62] | 59[50-65] | **<0.001** |
| 18-39 yrs | 13(6.5%) | 132(9.6%) |  |
| 40-64 yrs | 128(63.7%) | 957(69.5%) |  |
| >=65 yrs | 60(29.9%) | 288(20.9%) |  |
| **Comorbidities** |  |  |  |
| Diabetes | 69 (34.3%) | 278(20.2%) | **<0.001** |
| Hypertension | 84(41.8%) | 419(30.4%) | **0.001** |
| Dyslipidaemia | 14(7.0%) | 52(3.8%) | **0.035** |
| COPD | 5 (2.5%) | 45 (3.3%) | 0.55 |
| CKD | 1 (0.5%) | 3 (0.2%) | 0.46 |
| Heart failure | 8 (3.9%) | 40 (2.9%) | 0.40 |
| Smoking | 119(59.2%) | 539(39.1%) | **<0.001** |
| Physical Activity | 66(32.8%) | 635(46.1%) | **<0.001** |
| Family History | 47(23.4%) | 334(24.3%) | 0.787 |

Abbreviations: COPD: chronic obstructive pulmonary disease; CKD: chronic kidney disease; IQR: interquartile range; yrs: years
